# 2742. The Prevalence and Clinical Impact of Antibiotic Allergy Labels in Solid Organ Transplant Recipients

**DOI:** 10.1093/ofid/ofad500.2353

**Published:** 2023-11-27

**Authors:** Sashi Nair, Paul Bigliardi, Lauren Fontana

**Affiliations:** University of Minnesota, Minneapolis, Minnesota; University of Minnesota, Minneapolis, Minnesota; University of Minnesota, Minneapolis, Minnesota

## Abstract

**Background:**

Antibiotic allergy labels (AAL), specifically Penicillin allergy labels (PAL) are reported in up to 20% of immunocompromised patients. However, up to 95% of reported PALs can be disproven by skin and provocation testing. Compared to first line beta-lactam antibiotics, alternative agents are associated with significant complications, such as drug resistance, *C. diff* infection, and failure of antibiotics. Knowledge regarding the impacts of AAL on the Solid Organ Transplant (SOT) population is evolving. However, the impact on 1-year outcomes is not known. Our primary objective for this study was to evaluate the impact of AAL, particularly PALs on patient outcomes in SOT.

**Methods:**

We performed a retrospective review of adult SOT recipients who underwent a single kidney, liver, lung, heart, or pancreas (with or without a kidney transplant) transplant between 1/1/2012 and 1/1/2021. We subsequently identified recipients with at least 1 AAL at the time of transplant and collected clinical data,\ using our institutional database. Statistical analysis was performed with a Pearson’s chi-square test to assess association between categorical variables. Multivariate logistic regression was also performed on outcomes using significant variables from the univariate analysis.

**Results:**

2373 SOT recipients were included, and their details are shown in Table 1.In our cohort, 331(13.6%) had a PAL and 572 (24.1%) had at least one AAL. Lung recipients had a significantly higher rate of PAL than other organ types. Recipients with a PAL were statistically more likely to have pretransplant COPD, ESRD, or obesity (Table 2). Furthermore, recipients with a PAL were more likely to have graft failure and *C. diff, but* not death within the first year after transplant (Table 2). The association between PAL and *C. diff* rates persisted even in a multivariate model.

**Figure:**
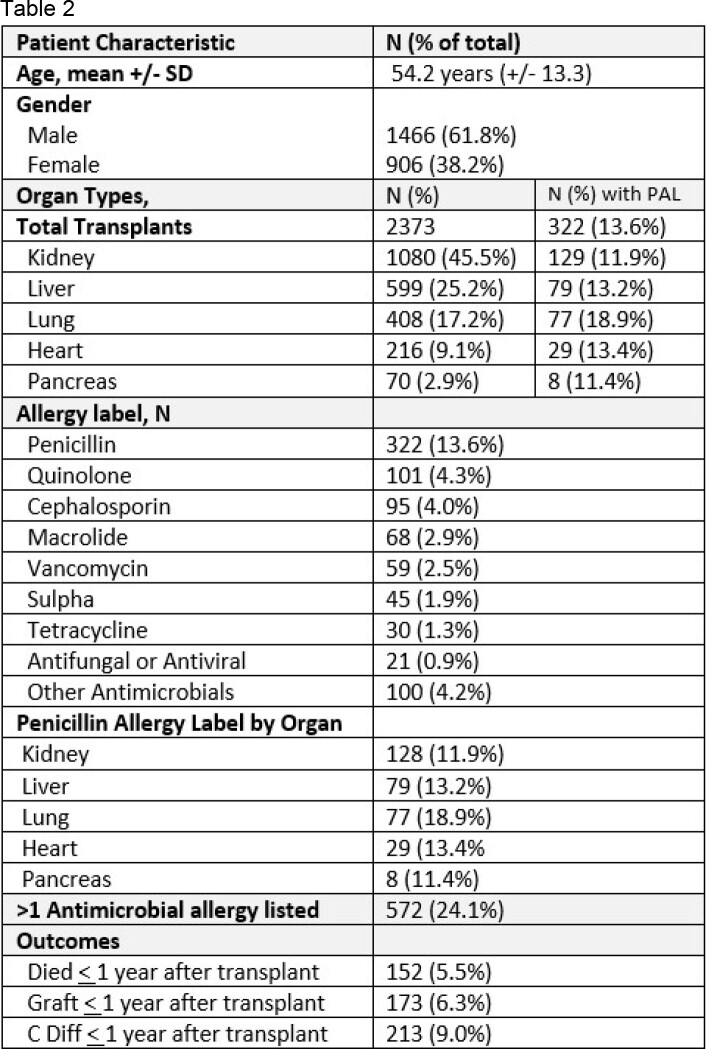

Univariate and Multivariate Logistical Regression Analysis for SOT Recipients with a Penicillin Allergy Label

**Figure:**
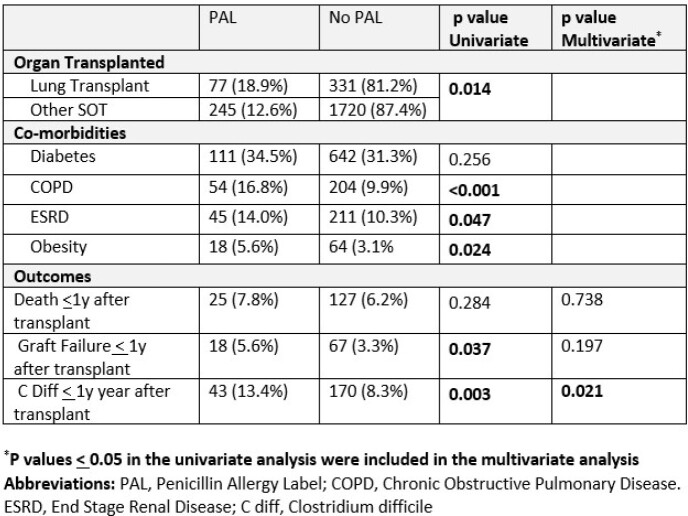

**Conclusion:**

To our knowledge this is the largest study with the longest duration of follow up evaluating the prevalence and outcomes of AAL and PAL in SOT recipients. Specifically, we identified worse outcomes associated with PAL such as higher C. diff rates and graft failure. This study highlights the need to better understand the impact of AAL and to promote multidisciplinary interventions that aim to remove invalid AAL pre-transplant.

**Disclosures:**

**All Authors**: No reported disclosures

